# Development and evaluation of a time-resolved fluorescence labelled immunochromatographic strip assay for rapid and quantitative detection of bovine herpesvirus 1

**DOI:** 10.3389/fmicb.2024.1371849

**Published:** 2024-02-29

**Authors:** Wenxiao Liu, Kun Zhang, Jing Cheng, Shiqiang Yu, Chunjie Cheng, Bo Jiang, Linyi Zhou, Yongqing Li

**Affiliations:** ^1^Institute of Animal Husbandry and Veterinary Medicine, Beijing Academy of Agriculture and Forestry Sciences, Beijing, China; ^2^Research Center for Infectious Diseases in Livestock and Poultry, Beijing Academy of Agricultural and Forestry Sciences, Beijing, China; ^3^Animal Science and Technology College, Beijing University of Agriculture, Beijing, China; ^4^Laboratory of Gastrointestinal Microbiology, Jiangsu Key Laboratory of Gastrointestinal Nutrition and Animal Health, National Center for International Research on Animal Gut Nutrition, College of Animal Science and Technology, Nanjing Agricultural University, Nanjing, Jiangsu, China

**Keywords:** bovine herpesvirus 1, diagnosis, lateral flow strip, time-resolved fluorescent microspheres, monoclonal antibody

## Abstract

Bovine herpes virus 1 (BoHV-1) causes a wide variety of diseases in wild and domestic cattle. The most widely used method for viral identification is real-time PCR, which can only be performed in laboratories using sophisticated instruments by expert personnel. Herein, we developed an ultrasensitive time-resolved fluorescence lateral flow immunochromatographic strip (ICS) assay for detecting BoHV-1 in bovine samples using a monoclonal antibody against BoHV-1 labelled with fluorescent microspheres, which can be applied in any setting. The intact process from sample collection to final result can be achieved in 15 min. The limit of detection of the assay for BoHV-1 was 10^2^ TCID_50_/100 μL. The coincidence rate of the ICS method and real-time PCR recommended by the World Organization for Animal Health (WOAH) was 100% for negative, 92.30% for positive, and 95.42% for total, as evaluated by the detection of 131 clinical samples. This detection method was specifically targeted to BoHV-1, not exhibiting cross-reactivity with other bovine pathogens including BoHV-5. We developed an ICS assay equipped with a portable instrument that offers a sensitive and specific platform for the rapid and reliable detection of BoHV-1 in the field. The Point-of-Care test of BoHV-1 is suitable for the screening and surveillance of BoHV-1 in dairy herds.

## 1 Introduction

Bovine herpes virus 1 (BoHV-1) infection can lead to various diseases in cattle, including infectious bovine rhinotracheitis and conjunctival infection, as well as abortion in cows and neurological disorders in calves (Jones and Chowdhury, 2007). Infected cattle may also develop secondary bacterial infections due to immunosuppression. Significant economic losses, due to growth retardation and significant reductions in milk production in affected livestock, have been attributed to BoHV-1 infections (Sayers, 2017). BoHV-1, a member of the genus Var*iellovirus* in the subfamily Alphaherpesvirinae, has three sub-types: BoHV-1.1, BoHV-1.2a, and BoHV-1.2b (Engels et al., 1986). BoHV-1.1 is the common cause of infectious bovine respiratory disease and abortions, whereas BoHV-1.2 causes genital tract infections. Subtype 1.1 strains have been more widely studied than 1.2 strains because they cause more serious diseases and appear to be more prevalent globally (Jones, 2019). Although animals infected with these three subtypes present different clinical signs, these strains share common antigenic properties (Meurens et al., 2004; Thiry et al., 2006).

Similar to other Alphaherpesvirinae viruses, BoHV-1 has a biphasic life cycle, with acute and latent infection phases. After acute infection, BoHV-1 can establish a lifelong latent infection in the trigeminal or sacral ganglia (Ostler and Jones, 2023). During latent infection, viral DNA in the neurons of the ganglia is below the limit of detection (LOD) (Grinde, 2013; Levings and Roth, 2013). Latent virus can be reactivated by treatment with the synthetic corticosteroid dexamethasone or by various stress factors, including transport or parturition. Therefore, convalescent cows can become carriers and transmit the BoHV-1 virus to the rest of the herd. Until recently, vaccination has been shown to be highly efficacious in reducing clinical signs. However, it cannot completely prevent viral transmission or reinfection. Further, recombination between the live attenuated vaccine and virulent strains can generate virulent recombinants (Lee et al., 2012). Therefore, adequate BoHV-1 infection control requires not only vaccination but also the application of diagnostic tests to identify virus carriers for culling.

Current diagnostic methods for BoHV-1 infection include serological tests, such as virus neutralization test (VNT) and ELISA, virus isolation and detection of viral DNA and antigens. Virus isolation is time-consuming and laborious, whereas techniques for viral DNA detection, such as PCR and real-time PCR, require dedicated instruments and are time-consuming as they require the extraction, amplification, and detection of DNA. As such, they are not suitable for large-scale sample detection or point-of-care testing (POCT) in cattle breeding or veterinary medicine. Therefore, a simple, rapid, and practical test for the identification and surveillance of BoHV-1 infections is needed. Immunochromatography assays (ICAs) are sensitive, specific, rapid, and easy to perform and have been widely applied in clinical diagnosis, food chemistry, and environmental monitoring (Tsuboi and Iinuma, 2021). Colloidal gold nanoparticles are widely used for commercial ICA test strips. To improve the sensitivity of ICAs, many other types of signal reporters have been applied, including magnetic nanoparticles, fluorescent nanoparticles, and chemiluminescent materials that are used in combination with a fluorescence signal reader (Huang et al., 2018; Ouyang et al., 2018; Li et al., 2020). Compared with traditional gold nanoparticle-based immunochromatographic strips (ICSs), they have improved sensitivity and quantitative capability.

In this study, we generated a series of highly sensitive monoclonal antibodies by immunizing BALB/c mice with BoHV-1 viruses. Based on these mAbs, we developed a new ICA platform using fluorescent nanoparticles coupled to BoHV-1-specific antibodies. Systematic evaluation indicated that this method can be applied for detecting BoHV-1 viruses in nasal, ocular, and genital swab samples. Combined with a field-portable equipment, it offers a specific and sensitive approach for rapid and quantitative detection of BoHV-1, especially in dairy farms with finite resources.

## 2 Materials and methods

### 2.1 Ethics statement

All animal experiments were performed in accordance with the guidelines of the Beijing Academy of Agricultural and Forestry Sciences Animal Care and Use Committee and were approved by the Animal Welfare Committee of the Beijing Academy of Agricultural and Forestry Sciences (December 15, 2017).

### 2.2 Virus, cells, and clinical samples

BoHV-1 Bartha Nu/67 was obtained from the China Veterinary Culture Collection Center (Beijing, China). BoHV-1.1 BJF03, BoHV-1.2 BJF16, BoHV-5 HBT12, bovine viral diarrhoea virus (BVDV) BJF19 and bovine coronavirus (BCoV) HBB07 were isolated by the Beijing Academy of Agriculture and Forestry Sciences. Bovine rotavirus (BRV) AV53 was purchased from the China Institute of Veterinary Drug Control (Beijing, China). Clinical samples suspected of BoHV-1, including cattle nasal and genital swabs, were collected from several dairy farms in Hebei and Beijing districts from 2022 to 2023. All samples were stored at −20°C.

### 2.3 Cell culture and virus purification

BoHV-1 and BoHV-5 isolates were grown in Madin–Darby bovine kidney (MDBK) cells (ATCC, Manassas, VA, United States) cultured in Dulbecco’s modified Eagle’s medium (DMEM; Gibco, Grand Island, NY, United States) supplemented with 10% fetal bovine serum (FBS; Gibco, Grand Island, NY, United States) at 37°C and 5% CO_2_. BVDV BJF19 was also grown in MDBK cells cultured in DMEM supplemented with 10% FBS at 37°C and 5% CO_2_. The BCoV isolate was grown in human rectal tumor (HRT-18) cells cultured in RPMI 1640 (Gibco, Grand Island, NY, United States) containing 5 μg/mL trypsin (Gibco). BRV AV53 was grown in MA-104 cells cultured in DMEM containing 5 μg/mL trypsin.

BoHV-1 virus was inoculated into MDBK cell monolayers in T75 flasks containing DMEM supplemented with 2% FBS and incubated for 2–3 d. BoHV-1-infected cells were harvested when 50–100% of cells showed cytopathic effects. The virus was isolated from cells by lysing the cells through three freeze–thaw cycles, followed by clarification with centrifugation at 3,000 × *g* for 30 min. The crude virus was concentrated and purified by sucrose density gradient centrifugation as previously described (O’Connor et al., 1964). Briefly, the virus was concentrated 100-fold via PEG6000 precipitation, layered onto a 100–400 mg/mL linear sucrose gradient and centrifuged at 30,000 rpm for 90 min at 4°C. The viral layer was collected using a sterile needle and transferred to polypropylene centrifuge tubes, where they were slowly diluted with 50 mM Tris–HCl (pH 8.0) and then centrifuged at 27,000 rpm for 3 h at 4°C. The supernatants were discarded, and the virus-containing pellets were re-suspended in 50 mM Tris–HCl.

### 2.4 Preparation and characterization of monoclonal antibodies against BoHV-1

Monoclonal antibodies (mAb) generated against BoHV-1 were prepared using purified BoHV-1 as the immunogen according to a previously described protocol (Liu et al., 2023). Briefly, mAbs were generated from ascites of BoHV-1-injected BALB/c mice with positive hybridoma cell lines and were purified using a HiTrap Protein A column (GE Healthcare, Memphis, TN, United States) according to the manufacturer’s instructions. Subsequently, mAb isotyping was performed using the IsoStrip kit (Sigma-Aldrich, St. Louis, MO, United States). The binding activity of mAbs to BoHV-1 was evaluated using an immunofluorescence assay. The specificity of mAbs was evaluated using western blotting and dot-blotting against BoHV-1, BVDV, BCoV, and BRV. ELISA additivity tests were utilised to identify whether mAbs recognised different BoHV-1 proteins and epitopes with reference to a previous study (Liu et al., 2023).

### 2.5 Coupling antibodies to time-resolved fluorescent microspheres

The purified mAbs were paired with an antibody conjugated to time-resolved fluorescent microspheres using immunochromatography, and another mAb was sprayed onto a nitrocellulose membrane. The mAbs conjugated to time-resolved fluorescent microspheres were prepared according to the manufacturer’s instructions (VDO Biotech, Suzhou, China). Briefly, the fluorescent microspheres were activated with 1-ethyl-3-(3-dimethylaminopropyl) carbodiimide hydrochlor (EDC) and N-hydroxysuccinimide (NHS) solutions. The activated microspheres were then coupled to mAbs in coupling buffer. Finally, the microsphere-coupled mAbs were blocked with blocking buffer and stored in storage buffer at 2–8°C away from light. Testing assays were performed by putting the nitrocellulose membrane in micro-centrifuge tubes containing the mixture of microsphere-conjugated mAbs and BoHV-1 antigen for 15 min; additional conjugate pads or other constitutions for lateral flow strips were not tested.

### 2.6 Development of BoHV-1 ICSs

**Figure 1:**
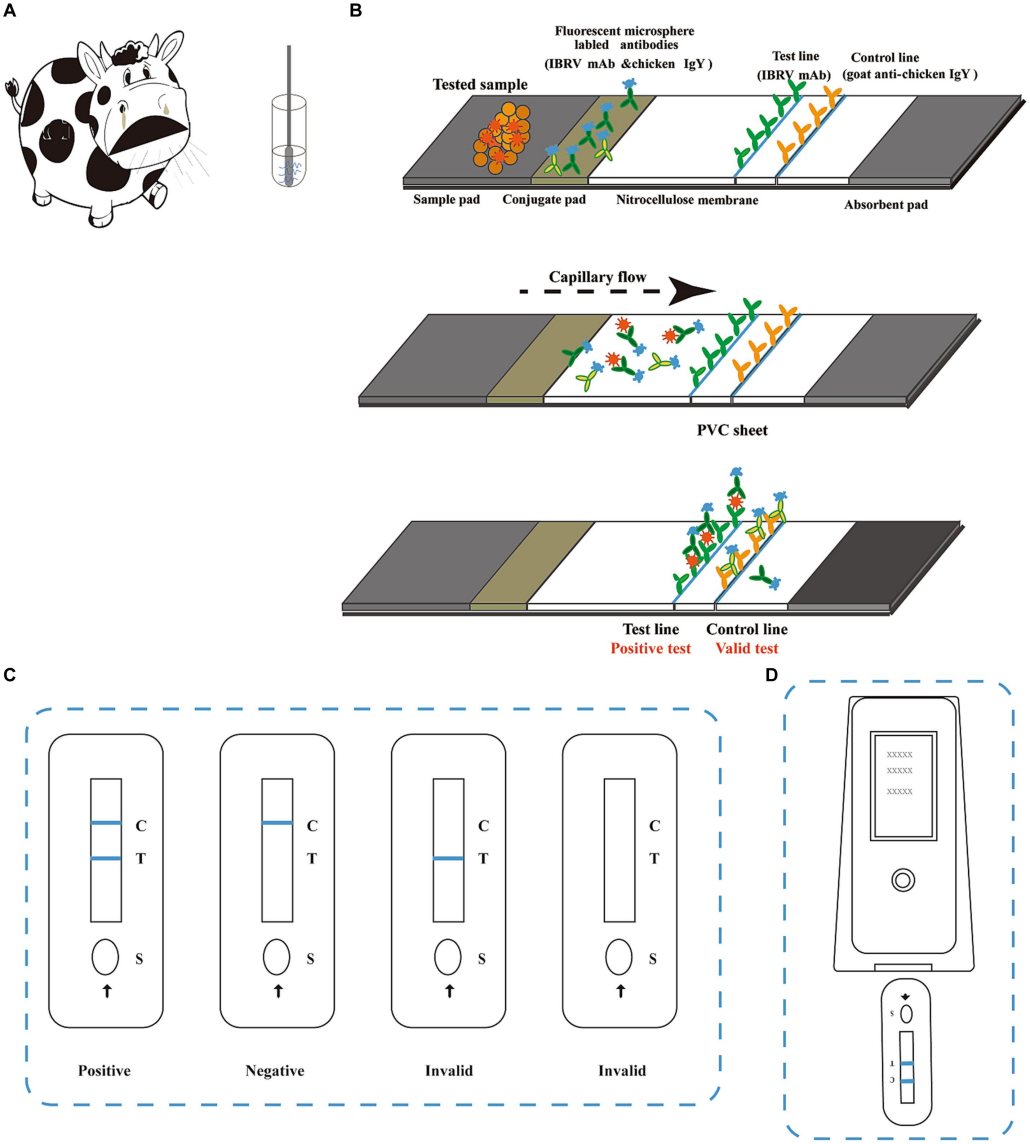
Schematic diagram of the BoHV-1 immunochromatographic strip assay. **(A)** Samples were either nasal swabs collected from bovines with respiratory disease or swabs of genital secretions or semen collected from bovines suspected of reproductive disease. For sample collection, the swab was inserted into the nostril of the bovine and slowly rotated over the surface of the nostril for 5 s to ensure its coverage with nasal fluid. After collection, the sample was inserted into a tube containing extraction buffer and rotated vigorously for at least 5 s. An 80 μL aliquot of the extracted sample was added to the sample well (S) on the strip. **(B)** The immunochromatographic strip (ICS) is comprised of a sample pad, conjugate pad, nitrocellulose membrane absorbent pad, and PVC sheet. A monoclonal antibody (mAb) targeting BoHV-1 (1B6) and chicken IgY were labelled with time-resolved fluorescent microspheres. The labelled antibodies were mixed and sprayed onto the conjugate pad. Another mAb targeting BoHV-1 (4F9) and goat anti-chicken IgY were placed on the nitrocellulose membrane at the test (T) and control (C) lines, respectively. BoHV-1 virus present in the test sample is captured by the antibodies conjugated to the time-resolved fluorescent microspheres and migrates along the membrane until it reaches the test line. Once the BoHV-1-mAb 1B6 complexes are captured by the other BoHV-1-specific antibody on the test line, the line becomes visible under UV light. The control line confirms that the sample has migrated across the membrane via capillary action. **(C)** Test results can be read under UV light at 10 min after adding the sample. Two distinct lines appearing on the strip, one bright blue line next to C and one bright blue line next to T, indicate BoHV-1 positive. Any faint line next to T should be considered as a positive result. Only one blue line next to C indicates a negative result. If no blue line is present next to C, the result is invalid, and the test should be repeated. **(D)** The results can be read using a fluorescent analyser to record the fluorescent signal on the test and control lines as the FI_T_ and FI_C_ values, respectively.

The ICS consisted of a conjugate pad, sample pad, nitrocellulose membrane, absorbent pad, and PVC sheet (Figure 1B). BoHV-1 mAb 1B6 conjugated to time-resolved fluorescent microspheres and time-resolved fluorescent microsphere-labelled chicken IgY were separately sprayed onto a glass cellulose membrane using an XYZ Platform dispenser HM3035 (Shanghai Kinbio Tech, Shanghai, China) at 4 μL/cm, and the conjugate pad was dried under vacuum. The 4F9 mAb and goat anti-chicken IgY (diluted to 0.5 mg/mL in phosphate buffered saline [PBS]) were used at the test and control lines, respectively, and were sprayed onto the nitrocellulose membrane using an XYZ large platform sensing dispenser HM3260 (Shanghai Kinbio Tech). Nitrocellulose membranes were then dried at 37°C overnight. All test components, including the sample pad, absorbent paper, pre-treated conjugate pad, and nitrocellulose membrane, were adhered to a PVC sheet (8 × 30 cm) in the proper order (Figure 1B). The sheets were then cut into 4-mm-wide strips using a programmable strip cutter (Shanghai Kinbio Tech). The strips were assembled in a plastic housing and stored at 25°C.

The lateral flow ICS was designed to detect extracted BoHV-1 antigens from bovine nasal, ocular or genital swabs (Figure 1). Prior to testing, the samples were eluted in extraction buffer (PBS with 0.5% Tween-20). The sample extract was then added to the sample well of the test strip. BoHV-1 present in the sample initially reacted with the mAb 4F9 conjugated to fluorescent microspheres in the glass fibre membrane. Then, the immune complex was captured by another anti-BoHV-1 mAb when it migrated to the test line on the nitrocellulose membrane. The results were observable under UV illumination within 10 min. Therefore, a positive reaction between BoHV-1 in the sample with the capture antibody should show as fluorescence on the test line. In contrast, a negative reaction, when the sample is free of BoHV-1, should only present a fluorescent signal on the control line due to the recognition of the chicken IgY by the goat anti-chicken IgY antibody conjugated with microspheres. BoHV-1 identified in the sample was quantitated using a fluorescent analyser (Laipson Technology, Luoyang, China).

### 2.7 Optimization and evaluation of the ICS assay

The optimal concentrations of the fluorescent microsphere-conjugated antibodies and capture antibodies were determined to maximise the signal intensity on the ICS. The BoHV-1 capture antibodies on the test (T) line and goat anti-chicken IgY on the control (C) line were applied at 0.2, 0.4, 0.6, 0.8, and 1 μg/cm. Different dilution buffers and reaction times were also used to optimise performance.

To assess the specificity for BoHV-1, closely related alphaherpesviruses and other bovine viruses, including BVDV, BCoV, BRV, BoHV-1 and BoHV-5 isolates were tested on the developed ICS. To assess the sensitivity, serial dilutions of purified BoHV-1 (10–10^7^ 50% tissue culture infectious dose [TCID_50_]/100 μL) in PBS were tested on the ICS. The virus was also serially diluted in semen to generate simulated samples. The LOD for the test strip was defined as the minimum dilution that produced a positive result. The reproducibility and stability of the ICS were evaluated by determining the average standard deviation of the fluorescent signals for BoHV-1-positive samples. All strips were stored at 25°C for 12 months and were evaluated at 3-month intervals.

### 2.8 Quantitative detection of BoHV-1 antigen using the ICS test

Approximately 30 μL of detected sample was premixed with BoHV-1 antigen and 60 μL sample dilution buffer at 25°C and added to the sample well of the pad. The strips were scanned using a fluorescent analyser after 10 min. The fluorescence signals of the T and C lines were recorded as FI_T_ and FI_C_, respectively, and the ratio of the fluorescence intensity of the test line to the fluorescence intensity of the control line (FI_T_/FI_C_) was calculated. For quantitative analysis, the amount of BoHV-1 was calculated using a linear regression equation for a BoHV-1 standard curve, which was generated by detecting BoHV-1 antigen with the following known concentrations of 0 (as negative control), 3.9 × 10^3^ (defined as std. 9), 7.8 × 10^3^ (std 8), 1.56 × 10^4^ (std 7), 3.12 × 10^4^ (std 6), 6.25 × 10^4^ (std 5), 1.25 × 10^5^ (std 4), 2.5 × 10^5^ (std 3), 5 × 10^5^ (std 2), and 10^6^ TCID_50_/100 μL (std 1). The experiment was repeated thrice.

### 2.9 Evaluation of the BoHV-1 ICS assay using clinical samples

To further evaluate the BoHV-1 ICS assay, 131 samples collected from dairy cows, including healthy and known BoHV-1-infected cows, were analysed using the BoHV-1 ICS and quantitative real-time PCR (qPCR) for BoHV-1 DNA in parallel. The qPCR assay was performed as previously described (Jiang et al., 2022). The FI_T_/FI_C_ for each sample as determined by the ICS assay was assessed using a fluorescence analyser. Receiver operating characteristic (ROC) curves were generated to determine the cut-off value using GraphPad Prism 8.0. All tests were repeated five times. The correlation coefficient of the BoHV-1 ICS versus qPCR was calculated by Pearson’s correlation coefficient analysis using GraphPad Prism 8.0.

## 3 Results

### 3.1 Purification of BoHV-1

Virus purity is important not only for the use of the virus as an immunogen for preparing mAbs but also for the standardization of the BoHV-1 ICS assay. BoHV-1 from our laboratory was propagated in up to 6 L of MDBK culture. Crude viruses were successfully concentrated and centrifuged in sucrose density gradients for 2 d. A visible opaque band was collected for purified BoHV-1 with a protein density of 27.9 mg/mL (Supplementary Table S1). The prepared BoHV-1 was analysed by PCR using specific primers targeting the gene encoding the viral protein gB. The obtained PCR product was sequenced and compared to the GenBank database using BLASTN, which showed that the queried sequence had 100% identity with the complete genome sequence of the BoHV-1 Bartha Nu/67 strain gB gene (GenBank ID: QBH74903.1). No non-specific immune response was observed in western blot analyses using mAbs against the BoHV-1 gD protein (Supplementary Figure S1).

### 3.2 Characterization of mAbs against BoHV-1

**Figure 2:**
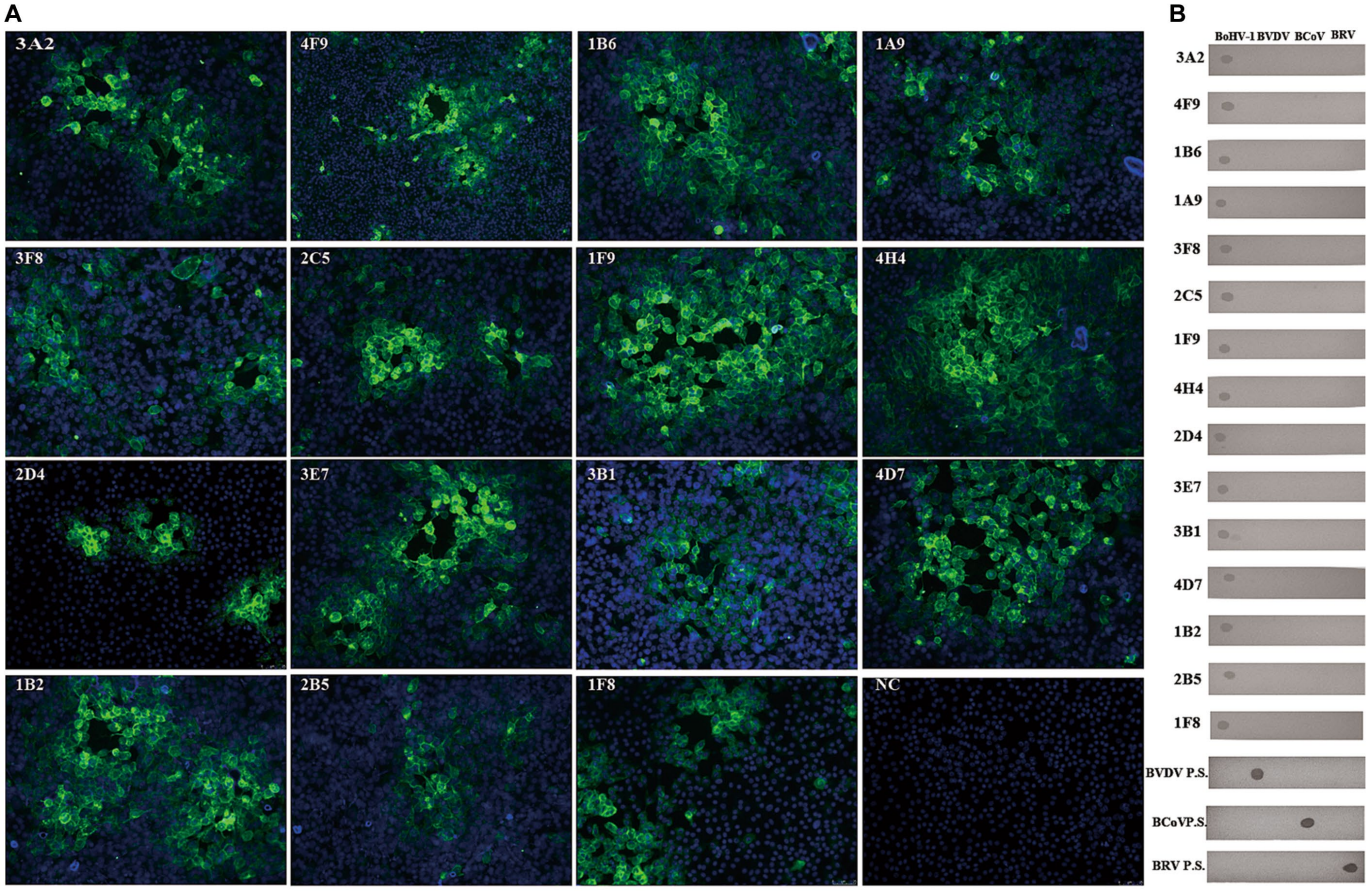
Generation and characterization of mAbs against BoHV-1. **(A)** Various isolated mAbs (*n* = 15) were used in indirect immunofluorescence assays on BoHV-1-infected MDBK cells (NC = negative control). **(B)** None of the BoHV-1 mAbs cross-reacted with BVDV, BCoV, or BRV, as determined by dot-blotting. The four inactivated viruses were added to nitrocellulose membranes, blocked with 3% BSA, and then incubated with mAb or virus-positive serum and HRP-conjugated goat anti-mouse IgG antibody or HRP-conjugated rabbit anti-bovine IgG, respectively. The blots were developed with DAB solution. (mAbs, monoclonal antibodies; BoHV-1, bovine herpesvirus 1; BD.P.S, positive serum samples for BVDV-specific antibody; BC.P.S, monoclonal antibody against BCoV; BR.P.S positive serum samples for BRV-specific antibody).

Using hybridoma antibody production, we obtained 15 monoclonal antibodies (mAbs; Table 1). These 15 mAbs were specific to BoHV-1 and did not cross-react with BVDV, BCoV, or BRV (Figure 2). ELISA revealed that four mAbs recognised gB, four recognised gD, and seven recognised gE. The titres of these mAbs in the ascites were > 1:1280000. Immunochromatography pair experiments revealed that mAb 1B6, which was used as the capture antibody, and mAb 4F9, which was used as the detectable antibody, were the most effective for the detection of BoHV-1 using the ICS assay. Sandwich ELISA further confirmed that the epitope recognised by mAb 1B6 was distinct from that recognised by 4F9. ELISA additivity tests indicated that the binding affinities of the two mAbs were additive.

**Table 1:** Characterization of monoclonal antibodies (mAb) against BoHV-1.

mAb name	Target protein	Subtype	Antibody titre in ascites
3A2	gB	IgG1, κ	1:128000
2C5	gB	IgG1, κ	1:128000
1F9	gB	IgG1, κ	1:256000
4H4	gB	IgG2b, κ	1:1024000
4F9	gD	IgG2b, κ	1:256000
1B6	gD	IgG1, κ	1:256000
1A9	gD	IgG1, κ	1:1024000
3F8	gD	IgG1, κ	1:512000
3A2	gE	IgG1, κ	1:1024000
2D4	gE	IgG1, κ	1:512000
3E7	gE	IgG1, κ	1:1024000
3B1	gE	IgG1, κ	1:256000
4D7	gE	IgG2b, κ	1:1024000
1B2	gE	IgG2b, κ	1:512000
2B5	gE	IgG1, κ	1:128000
1F8	gE	IgG1, κ	1:256000

### 3.3 Development and optimization of the BoHV-1 ICS assay

Antibodies labelled with fluorescent microsphere are critical components of the BoHV-1 ICS assay protocol. Purified mAb 1B6 and goat anti-chicken IgY were individually labelled with fluorescent microspheres prior to testing. As shown in Figure 1, for samples containing BoHV-1, fluorescence was detected at both the test and control lines, whereas for mock samples, fluorescence was only detected at the control line (goat anti-chicken IgY antibody conjugated with fluorescent microspheres with chicken IgY). To optimise the assay, the concentration of the labelled antibodies was varied to enhance the BoHV-1 detection sensitivity. The strongest signal was observed at concentrations of mAb 4F9 and goat anti-chicken IgY of 0.4 and 0.8 μg/cm, respectively. The optimal concentration of mAb 1B6 in the reaction mixture sprayed on the glass cellulose membrane was 80 μg/mL.

### 3.4 Cut-Off value, cross-reactivity, sensitivity, and stability of the BoHV-1 ICS assay

**Figure 3:**
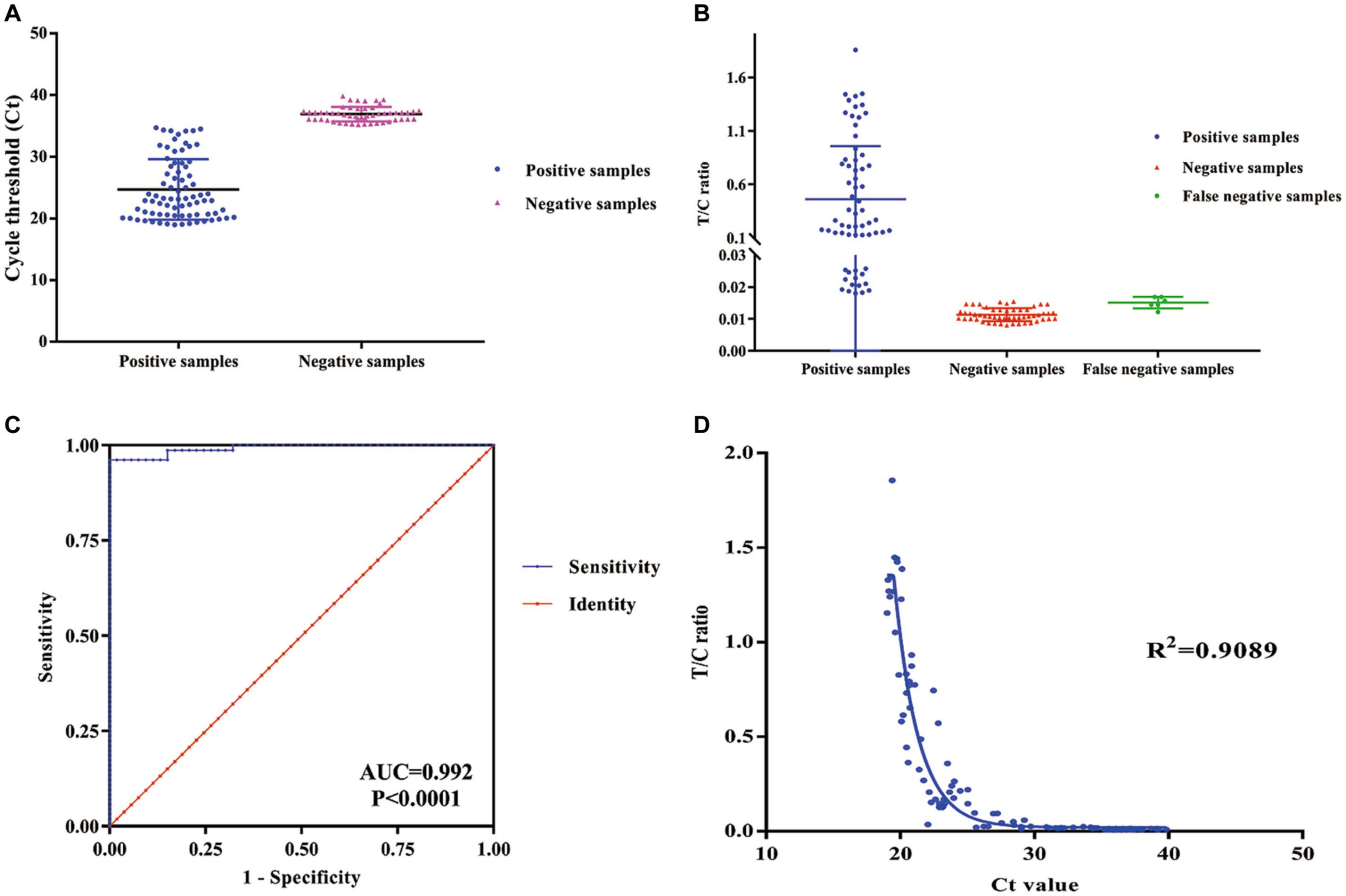
ROC curve analysis to determine the cut-off value, specificity, and sensitivity of the ICS assay. **(A)** qPCR cycle threshold Ct values for positive and negative samples. **(B)** FI_T_/FI_C_ values for positive, negative, and false negative samples as determined using the ICS assay. **(C)** ROC curve analysis was carried out to determine the cut-off of the ICS assay. **(D)** The sensitivity and specificity of the ICS assay was determined using ROC curve analysis. (ROC, receiver operating characteristic; qPCR, quantitative reverse transcription PCR; ICS, immunochromatography strip).

**Figure 4:**
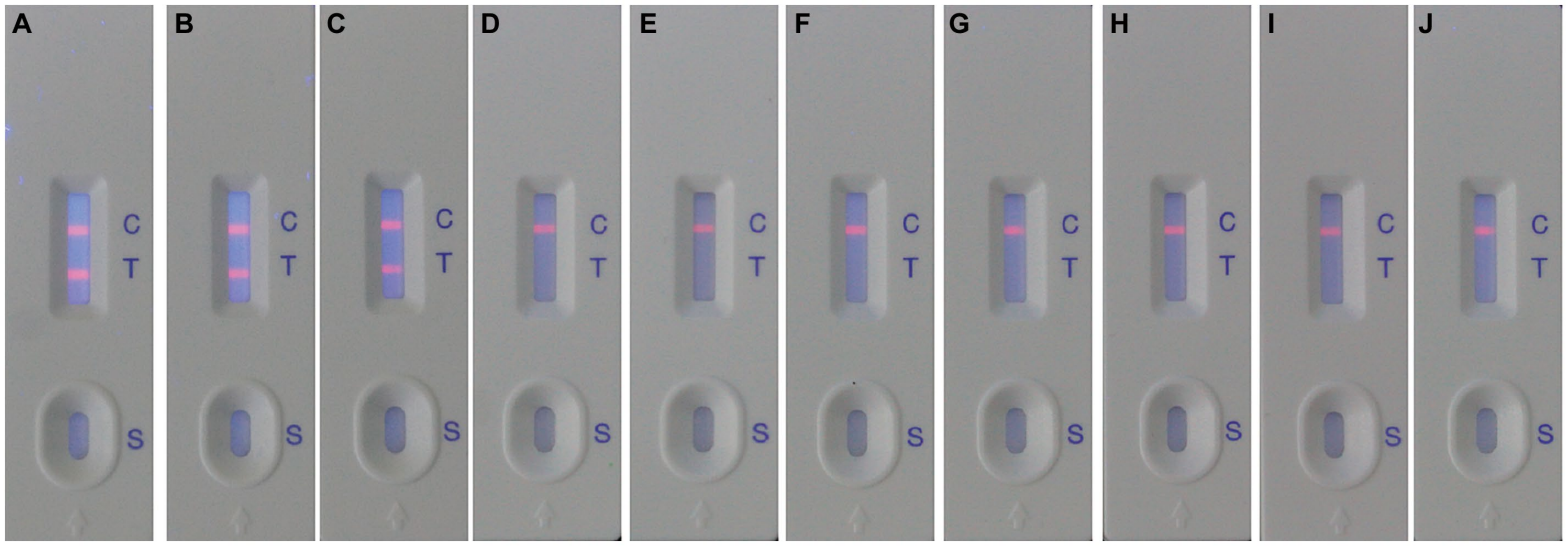
Evaluation of the specificity of the ICS for detecting BoHV-1. The following viruses were detected using the ICS assay: BoHV-1 Bartha Nu/67 (strip A); BoHV-1.1 BJF03 (strip B); BoHV-1.2 BJF16 (strip C); BoHV-5 HBT12 (strip D); BVDV BJF19 (strip E); BCoV HBB07 (strip F); BRV AV53 (strip G); MDBK (control, strip H); HRT-18 (control, strip I); MA104 (control, strip J).

**Figure 5:**
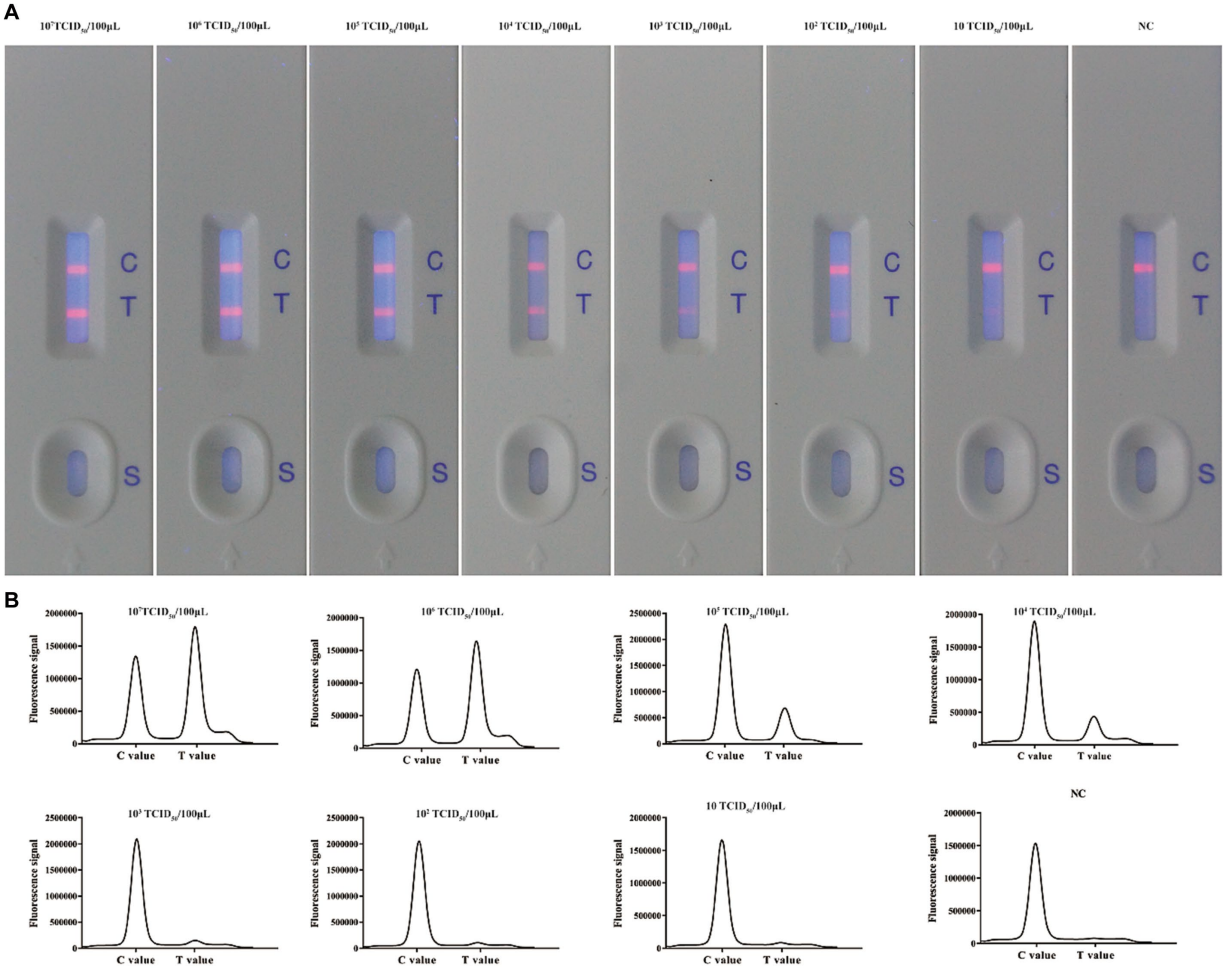
Evaluation of the sensitivity of the ICS for detecting BoHV-1. **(A)** Ten-fold dilutions of a BoHV-1 sample were tested using the ICS assay. The coloured line next to the test line (T) gradually became fainter as the concentration of virus decreased. **(B)** Absorption peaks of samples with different concentrations of BoHV-1.

After optimizing the BoHV-1 ICS assay, the cut-off value was determined using 78 samples from BoHV-1-infected Holstein cows and 53 samples from healthy bovines. qPCR analysis showed that the 78 samples were identified as BoHV-1 positive, with cycle threshold (Ct) values ranging from 22 to 35. The viral load in each sample was measured using both the BoHV-1 ICS and real-time PCR (Figures 3A,B). The area under the ROC curve (AUC) for the BoHV-1 ICS assay was 0.992 (standard error [SE] = 0.005343), with a 95% CI of 0.9815–1.002 (Figure 3C). ROC curve analysis indicated that the cut-off value for the ICS assay was 0.01886 (Figure 3D), with optimal specificity of 0.9811 and sensitivity of 0.9615. The coincidence rate of the ICS method and real-time PCR was 100% for negative, 92.30% for positive, and 95.42% for total, as determined by the evaluation of 131 clinical samples (Table 2). To evaluate the cross-reactivity of the assay, various pathogenic bovine viruses, including BoHV-1 Bartha Nu/67 and BJF03, BVDV BJF19, BRV, BCoV, and BoHV-5 HBT12, were tested using the assay. While a positive signal was detected for all BoHV-1 viruses, negative results were obtained for all other tested viruses including BoHV-5 (Figure 4). Only samples containing BoHV-1 were positive, indicating that the BoHV-1 ICS assay can distinguish BoHV-1 from other bovine viruses (Table 3). Therefore, the BoHV-1 ICS assay was demonstrated to have good specificity. Next, we determined the LOD of the BoHV-1 ICS assay. Samples containing various titres of BoHV-1 were placed in the sample well of the optimised ICS. The results showed that the LOD was 100 TCID_50_/100 μL (Figure 5). The LOD of the ICS assay using different simulated samples, including cow respiratory and reproductive tract secretions and semen was also determined. The results showed that the BoHV-1 titres in reproductive tract secretions detected by ICS were similar to those detected in PBS. However, bovine semen containing BoHV-1 migrated more slowly, and the fluorescence signal of the test line was weaker than that of tested sample dilutions. The LOD for semen samples was 1,000 TCID_50_/100 μL.

**Table 2:** Comparison of the results of the ICS and qPCR for 131 tested samples from healthy and known BoHV-1-infected cows.

		Real-time PCR		
		Negative	Positive	Total
	Negative	53	6	59
ICS	Positive	0	72	72
	Total	53	78	131
Coincidence rate				95.42%

**Table 3:** Cross-reactivity of the ICS against common bovine pathogens.

Sample source	No. of samples	Diagnostic performance		
		P	D	N
BoHV-1-infected bovines	5	5	0	0
BoHV-5-infected bovines	5	0	0	5
Bovine viral diarrhoea virus (BVDV)-infected bovines	5	0	0	5
Bovine rotavirus (BRV)-infected bovines	5	0	0	5
Bovine coronavirus (BCoV)-infected bovines	5	0	0	5

*P* positive, *D* doubtful, *N* negative.

**Figure 6:**
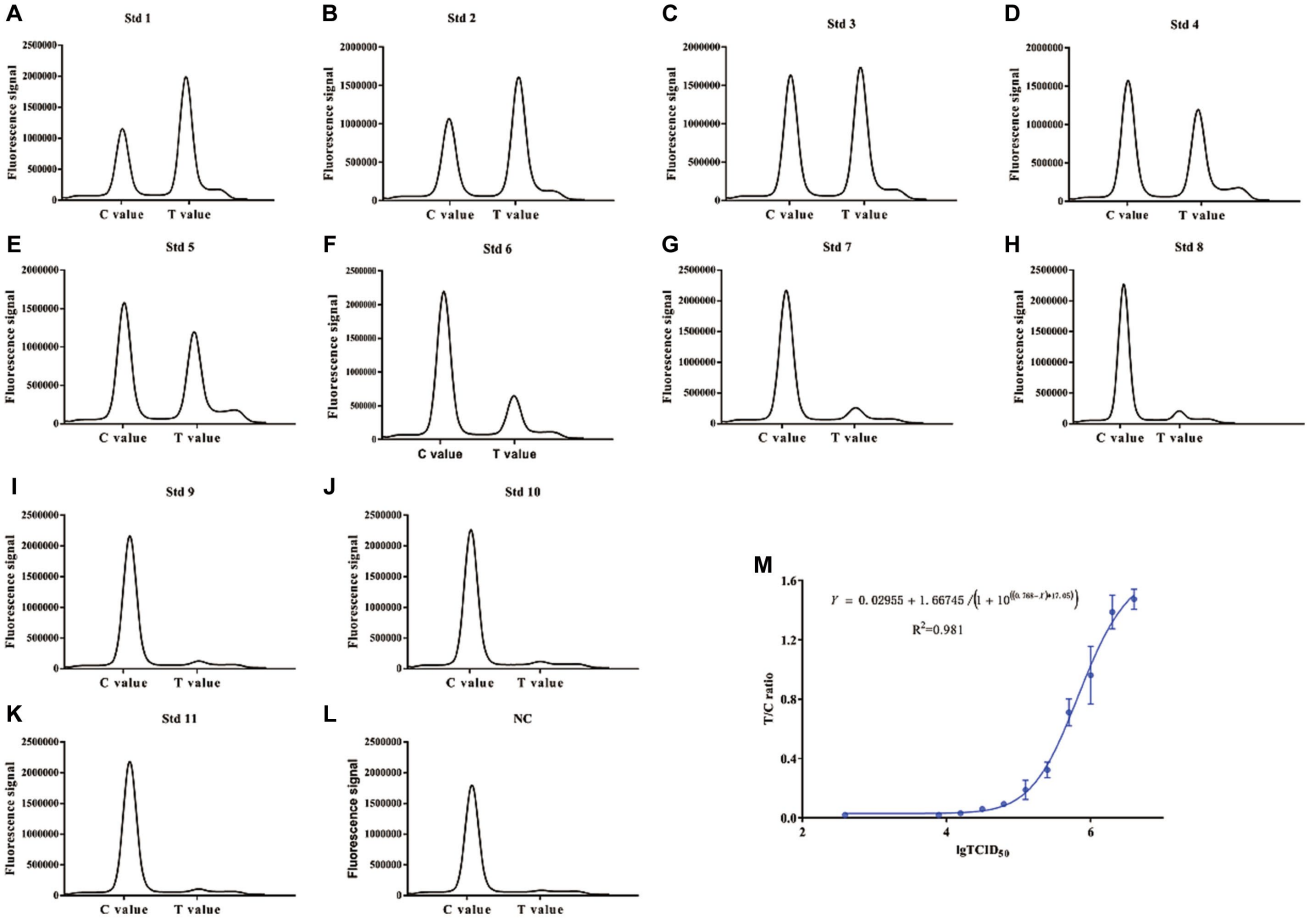
Correlation between FI_T_/FI_C_ values and the 50% tissue culture infectious dose (TCID_50_). The two fold diluted BoHV-1 samples were tested using both the ICS and TCID_50_ assays. **(A–L)** Absorption peaks recorded by the fluorescent analyser. **(M)** Plot of the FI_T_/FI_C_ values and TCID_50_.

Based on our established method, tested samples were spiked with BoHV-1 virus at different concentrations (0, 3.9 × 10^3^, 7.8 × 10^3^, 1.56 × 10^4^, 3.12 × 10^4^, 6.25 × 10^4^, 1.25 × 10^5^, 2.5 × 10^5^, 5 × 10^5^, and 10^6^ TCID_50_/100 μL) and detected using ICS combined with a portable electric device. Our results indicated that the absorbance peak of T lines became higher with increasing BoHV-1 concentrations. On the contrary, the absorbance peak of T lines became lower until disappeared with decrease concentration of BoHV-1 (Figures 6A–L). The calibration curve was generated based on the FI_T_/FI_C_ (the ratio of peak heights of T to C lines) according to the known concentrations of determined BoHV-1 viral samples. As shown in Figure 6M, when the FI_T_/FI_C_ was greater than 0.0316 or the viral titre in samples was greater than 10^4^ TCID_50_/100 μL, the T/C values were well correlated with the viral TCID_50_ (R^2^ = 0.981). This suggested that this fluorescence-based method can be applied for the quantitative measurement of virus in test samples.

To evaluate the stability of the ICS during storage, three batches of BoHV-1 ICSs were produced and used to detect BoHV-1 in clinical samples. As shown in Table 4, different batches of strips stored at 25°C for 3, 6, and 9 months showed identical performance for the detection of BoHV-1 samples. However, after storage at 25°C for 12 months, seven of the 20 tested positive samples were determined as being negative; indicating that the maximum storage period for the ICS at 25°C is 9 months.

**Table 4:** Stability and reproducibility of BoHV-1 detection using the ICS assay.

Batch	F210615				F210630				F230715			
Storage time (months)	3	6	9	12	3	6	9	12	3	6	9	12
Positive (no.)	15	15	15	13	15	15	15	14	15	15	15	13
Negative (no.)	5	5	5	7	5	5	5	6	5	5	5	7

### 3.5 Evaluation of the BoHV-1 ICS for the testing of clinical samples

**Figure 7:**
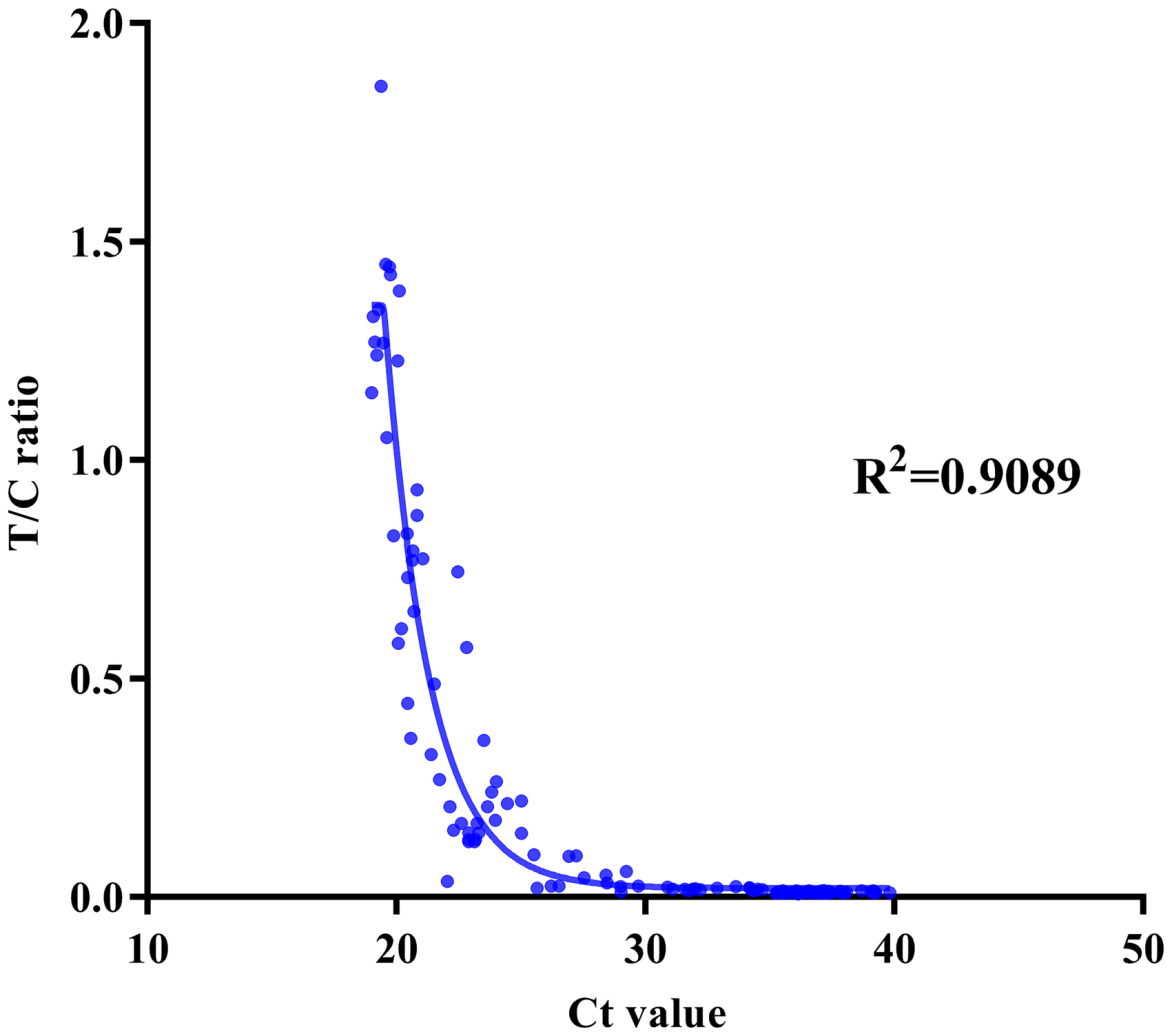
Correlation between the FI_T_/FI_C_ (T/C) ratio as determined by the ICS assay and the cycle threshold (Ct) value as determined by quantitative reverse transcription PCR.

To evaluate the clinical application of the BoHV-1 ICS assay, 137 clinical samples were collected from dairy farms and simultaneously tested using both the BoHV-1 ICS and qPCR assays. qPCR analysis showed that 28 bovine nasal swab samples (P1–28) were BoHV-1 positive, with cycle threshold (Ct) values ranging from 22 to 32, while 15 bovine semen samples (P29–43) were BoHV-1 positive, with Ct values ranging from 25 to 32. Other tested samples were BoHV-1 negative, including 23 nasal swab samples (S1–23) from cows with fever and cough, 20 nasal samples (S24–43) from bovines infected with BVDV or BCoV, 30 nasal samples from healthy bovines (S45–74), and 20 semen samples (S75–94) from healthy bovines (Figure 3A). All but one of the qPCR-positive samples (P1–43) tested positive for BoHV-1 with the ICS assay, with FI_T_/FI_C_ values of 0.1–0.90. The sample that tested negative with BoHV-1 ICS was also BoHV-1 negative when using virus isolation. All PCR-negative samples were also BoHV-1 negative when tested using the ICS assay. Comparison to the qPCR results showed that the BoHV-1 ICS had a sensitivity of 99.01%. The correlation between the FI_T_/FI_C_ values determined by ICS and Ct values determined by qPCR was 0.9089 (Figure 7).

## 4 Discussion

Pathogen diagnosis is the first step in the control and prevention of infectious diseases in beef and dairy farms. Infectious bovine rhinotracheitis mainly caused by BoHV-1 is a highly contagious disease, resulting in significant economic losses to the farms (Righi et al., 2023). As BoHV-1 can become latent within 1–6 d after primary infection, rapid, sensitive, and specific diagnostic techniques are needed for the prevention, control, and eradication of BoHV-1 infections (Zhao J. et al., 2020; Ostler and Jones, 2023). Although several diagnostic techniques for BoHV-1 infection are available, including cell culture, histopathology, serology, and real-time PCR, none can rapidly or quantitatively detect the virus on site. This study demonstrated the feasibility of a POCT method, namely, an ICS labelled with fluorescent nanoparticles, for detecting BoHV-1 in nasal cavity swabs and semen within 10 min. This method was mainly designed for the diagnosis of BoHV-1, which is responsible for the respiratory syndrome and genital infections in bovines. Other subtypes were not monitored in this study. When combined with a fluorescence signal reader, this method can quantitatively measure viral loads in samples. Such rapid and accurate detection will be helpful for controlling and eradicating BoHV-1 infections.

Various test methods have been developed for the diagnosis of BoHV-1, including loop-mediated isothermal amplification assay, duplex real-time PCR and droplet digital PCR assays (Marin et al., 2016; Peltzer et al., 2021; Ali et al., 2022; Yu B. et al., 2022; Yu Z. et al., 2022). Both droplet digital PCR assays and loop-mediated isothermal amplification are very sensitive methods for the diagnosis of BoHV-1 infections; however, these methods can only be performed by personnel with technical expertise in well-equipped laboratories. Following the outbreak of infectious disease in farms, farmers have to collected samples and send them to registered laboratories. Taking into consideration the process of nucleotide acid extraction, it might take a day or two or even longer after the samples have reached the lab for obtaining results. However, the period of acute BoHV-1 infection is 7–10 d resulting in high levels of viral shedding in oral, nasal, ocular, or genital cavities (Jones, 2019). Viral shedding in the semen may differ among BoHV-1-infected bulls and although the viral titre will not be very high, 200 TCID_50_ of virus in artificial insemination can initiate BoHV-1 infection (Van Oirschot, 1995). Consequently, a definite diagnosis as early as possible is optimal for reducing the risk of transmission. On-site detection is the future trend for pathogen diagnosis, because it is simple, rapid and user-friendly (Zhao V. X. T. et al., 2020; Xiao et al., 2022; Hu et al., 2023; Seok et al., 2023). It has been widely used in many fields of research. However, our study is the first to establish a BoHV-1 ICS assay with several advantages over traditional methods. First, the BoHV-1 ICS assay has reduced workflow and testing time, resulting in more rapid diagnosis. Second, the BoHV-1 ICS assay does not require sophisticated sample preparation, precise instruments, or well-equipped laboratories, allowing farmers to perform the test in any setting. Furthermore, it can eliminate false-positive results due to aerosol contamination as no amplification is performed. Finally, the viral load in samples can be quantified based on the signal intensity of the test line (Pawar et al., 2014).

Recently, the conventional ICS method has been improved to achieve higher sensitivity and specificity using biosensors and antibodies (Yu B. et al., 2022; Yu Z. et al., 2022). These studies have been applied for developing a fluorescence immunochromatographic (FIC) assay to detect SARS-CoV-2 (Diao et al., 2021). The specificity and sensitivity of the FIC assay were 100 and 75.6%, respectively, which were comparable to those of real-time PCR. Liu et al. (2020) developed a novel ICA based on ultramarine blue particles to detect hepatitis B virus, with an LOD of 0.37 ng mL^−1^. In this study, we generated many mAbs against BoHV-1 using the hybridoma cell fusion technique. To improve the sensitivity and specificity, we selected two mAbs that recognise non-overlapping regions of a viral antigen. Previous studies demonstrated that specific mAbs combined with time-resolved fluorescent microspheres as a biosensor can not only reduce background interference but also increase the sensitivity of detection compared with traditional signals (Juntunen et al., 2012; Hu et al., 2021; Wu et al., 2021). The LOD of our developed test strip was 100 TCID_50_/100 μL in PBS and 1,000 TCID_50_/100 μL in simulated semen samples. The sensitivity of the developed test strip was lower than that of droplet digital PCR or real-time PCR. The negative coincidence rate, positive coincidence rate and accuracy were compared to verify the feasibility of the developed method in this study. Our results confirmed that the developed ICS showed high negative coincidence rate and accuracy comparable to those of traditional methods. In addition, the developed method was convenient and suitable for on-site quick inspection by veterinarians in dairy farms. Furthermore, based on the established calibration curves, the operator is capable of quantifying the concentration of BoHV-1 virus in tested samples. Fast screening the infected bovine that is at the stage of lytic BoHV-1 infection is particularly helpful. In such cases, POCT is more suitable than qPCR. In addition, the mAbs generated in this study were specifically targeted to BoHV-1, and showed no cross-reactivity with other viruses. Further experiments confirmed that our strips did not cross-react with other tested bovine pathogens, including BVDV, BCoV, and BRV. Most importantly, our developed method showed high conformance with the gold-standard method of qPCR in the case of clinical samples, demonstrating that FI_T_/FI_C_ correlated with the viral load. These results indicated that BoHV-1 ICS can be used to rapidly screen BoHV-1-positive cattle in the field, especially those with respiratory symptoms.

In conclusion, we successfully developed a time-resolved fluorescent immunochromatographic assay for the quantitative detection of BoHV-1 antigens with high sensitivity, strong specificity, and good reproducibility and stability that offers a POCT tool to rapidly screen and eliminate BoHV-1-positive cattle. The diagnostic method developed in this study would be appropriate for on-site detection of BoHV-1, facilitating the efficient control and reducing the transmission of BoHV-1.

## Data availability statement

The original contributions presented in the study are included in the article/Supplementary material, further inquiries can be directed to the corresponding author.

## Ethics statement

The animal study was approved by Beijing Academy of Agricultural and Forestry Sciences Animal Care and Use Committee. The study was conducted in accordance with the local legislation and institutional requirements.

## Author contributions

WL: Conceptualization, Formal analysis, Funding acquisition, Methodology, Project administration, Writing – original draft, Writing – review & editing. KZ: Data curation, Investigation, Methodology, Software, Writing – review & editing. JC: Formal analysis, Project administration, Software, Visualization, Writing – review & editing. SY: Data curation, Methodology, Software, Writing – review & editing. CC: Formal analysis, Investigation, Supervision, Visualization, Writing – review & editing. BJ: Investigation, Supervision, Validation, Writing – review & editing. LZ: Investigation, Methodology, Project administration, Resources, Writing – review & editing. YL: Funding acquisition, Investigation, Software, Writing – review & editing.
