# Supplementary material for: Development and evaluation of a time-resolved fluorescence labelled immunochromatographic strip assay for rapid and quantitative detection of bovine herpesvirus 1
Source: Front Microbiol. 2024 Feb 29;15:1371849. doi: 10.3389/fmicb.2024.1371849 (PMC10937450; doi:10.3389/fmicb.2024.1371849)

**Supplementary material**

**Table S1. Concentration and purification of BoHV-1 by sucrose density gradient centrifugation**

| Virus | Volume (mL) | Protein total (mg) |
| --- | --- | --- |
| Crude BoHV-1 | 3,000 | 8,256 |
| Concentrated BoHV-1 | 67 | 423 |
| Purified BoHV-1 | 4 | 111.6 |

**Figure S1. Identification of purified BoHV-1 using western blotting.** MDBK cells were infected with BoHV-1. Crude BoHV-1 was collected and purified by sucrose-density gradient centrifugation. Western blotting was performed to detect β-actin and gD using a β-actin antibody and an anti-gD monoclonal antibody (3F8), respectively. (pBoHV-1, purified BoHV-1; cBoHV-1, crude BoHV-1.)


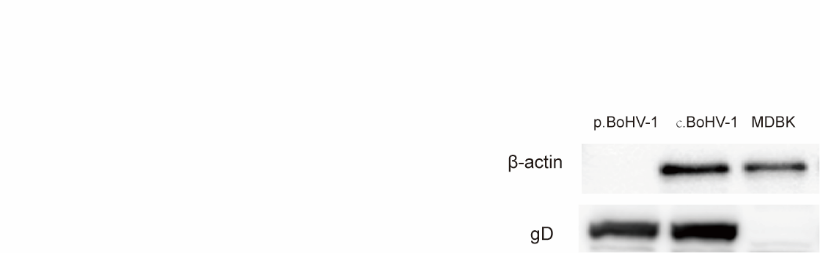

Supplement: Supplementary file 1 [file Data_Sheet_1.docx]
